# Therapeutics Targeting p53-MDM2 Interaction to Induce Cancer Cell Death

**DOI:** 10.3390/ijms23095005

**Published:** 2022-04-30

**Authors:** Nayeong Koo, Arun K. Sharma, Satya Narayan

**Affiliations:** 1 Department of Anatomy and Cell Biology, University of Florida, Gainesville, FL 32610, USA; nayeongkoo@ufl.edu; 2 Department of Pharmacology, Penn State Cancer Institute, College of Medicine, Penn State University, Hershey, PA 17033, USA; aks14@psu.edu

**Keywords:** p53, Mdm2, cancer, small molecules, chemotherapy

## Abstract

Named as the guardian of the genome, p53 is a tumor suppressor that regulates cell function, often through many different mechanisms such as DNA repair, apoptosis, cell cycle arrest, senescence, metabolism, and autophagy. One of the genes that p53 activates is MDM2, which forms a negative feedback loop since MDM2 induces the degradation of p53. When p53 activity is inhibited, damaged cells do not undergo cell cycle arrest or apoptosis. As 50% of human cancers inactivate p53 by mutation, current research focuses on reactivating p53 by developing drugs that target the p53-MDM2 interaction, which includes the binding of MDM2 and phosphorylation of p53. The objective of this article is to provide a short list and description of p53-MDM2 antagonists that may be excellent candidates for inducing cancer cell death. Relevant articles were searched for and identified using online databases such as PubMed and ScienceDirect. Increasing p53 levels, by targeting the p53-MDM2 interaction, can help p53 play its role as a tumor suppressor and induce cancer cell death. Researchers have identified different compounds that can act as inhibitors, either by directly binding to MDM2 or by modifying p53 with phosphorylation. The results associated with the drugs demonstrate the importance of targeting such interactions to inhibit cancer cell growth, which indicates that the use of the compounds may improve cancer therapeutics.

## 1. Introduction

P53 is also known as tumor suppressor protein p53 (TP53) and the guardian of the genome. It is a 393 amino acid transcription factor that regulates cell cycle progression. It can act as a transcriptional activator or repressor, which often leads to tumor suppression [1]. Depending on cellular stresses and cell cycle conditions, p53 can lead to various biological responses [2,3]. For example, if DNA damage is present, p53 is activated to transcribe target genes, such as *Puma* and *Btg2*, which induce DNA repair, apoptosis, cell cycle arrest, and senescence [4]. In addition, p53 is involved in metabolism and autophagy [5,6,7]. For instance, p53 prevents the metabolic reprogramming of cancer cells [7]. Moreover, autophagy, which can inhibit tumorigenesis by the engulfment and degradation of damaged cellular organelles, is promoted through the activation of p53-induced target genes, including *Atg10* [1]. Thus, there are several ways by which p53 regulates cell function. As the guardian of the genome, p53 regulates cell proliferation and acts as a tumor suppressor. Since p53 is a highly studied molecule, there is a vast repository of literature on this topic. We have tried to cite the most relevant references in this review. Our focus has been to describe some small molecules which have the potential for further development as therapeutic agents or that serve as leads for developing clinically viable agents targeting p53 and mouse double minute 2 homolog (MDM2) interaction. Thus, the entire review is organized from this point of view.

**Figure 1:**
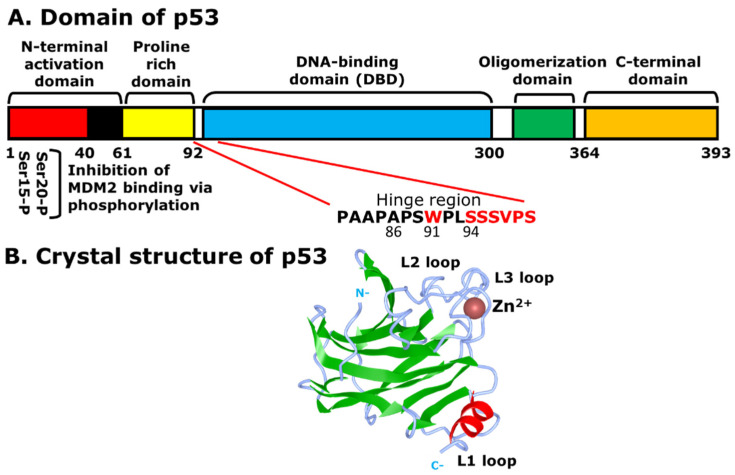
Structural features of p53: (**A**) different domains of p53. The Ser-15 and Ser-20 of the N-terminal activation domain (1–61), or TAD1 and TAD2, undergo phosphorylation and are the specific sites for interaction with MDM2 for ubiquitination and degradation of p53, although Mdm2 can bind to other domains, such as the C-terminal domain. Other domains include the proline rich domain (64–92), the DNA-binding domain (DBD) (100–300), the oligomerization domain (323–355), and the C-terminal domain (364–393); (**B**) the DNA-binding domain (DBD) of p53 with the absence of DNA. The bound zinc ion is shown as a brown sphere. The β-sheet and α-helix are displayed in green and red, respectively. The N-terminal activation domain (N) and the C-terminal domain (C) are labeled in grey.

As the name indicates, the molecular size of p53 is 53 kilodaltons (kDa), containing the N-terminal activation domain (1–61) or the transactivation domain (TAD), the proline-rich domain (64–92), the DNA-binding domain (DBD) (100–300), the oligomerization domain (323–355), and the C-terminal domain (364–393) (Figure 1A). The N-terminal activation domain consists of two subdomains, TAD1 (1–40) and TAD2 (40–61), which interact with MDM2 for the proteasomal degradation and regulation of p53 [8]. TAD1 plays a more important role in transactivation than TAD2, as it is required for the induction of cell cycle arrest at G_1_ and apoptosis, although each TAD is specialized to transactivate different target genes and pathways [9]. The second domain on p53 is the proline-rich domain, which does not have a functional effect on the transactivation of MDM2. However, the proline-rich domain is positioned in such a way that it moves the TAD away from the DBD, and indirectly affects apoptosis due to an alteration of transcriptional repression [10,11]. The DBD, on the other hand, is a core domain that recognizes specific DNA sequences, such as 5′-PuPuPuC(A/T)(T/A)GPyPyPy-3′, in which Pu and Py indicate purine and pyrimidine bases, respectively [9]. Located between amino acid residues 100–300, the DBD is folded into a β-sheet, which contains short α-helices, and has two inverted sequences that bind to the consensus DNA as response elements (REs) [12]. It also has a zinc ion (Zn^2+^) near the DNA-binding area that is essential for site-specific DNA-binding, which provides stability for the DBD [13,14]. Without Zn^2+^, the DBD loses the site-specific DNA-binding ability, although it retains non-specific binding, and decreases the overall stability and transcriptional activity of p53 (Figure 1B) [15]. The Arg248 of the L3 loop of p53 binds to the DNA minor groove directly or through water-mediated contacts with the DNA backbone. It has been found that one structural water molecule can form four hydrogen bonds with the DNA backbone, the main-chain of Cys277, the side-chain of Arg280, and the side-chain of Asp281, which in turn becomes stabilized via a salt-bridge network with the guanidinium groups of Arg273 and Arg280 [16,17]. Another important structural feature of p53 is the presence of a hinge region between the DBD and the proline-rich region at the N-terminus. The hinge region expands between 86–93 amino acids of the DBD and the proline-rich region (Figure 1A) [10]. The hinge region plays an important role in differentially regulating p53-mediated cell cycle arrest and apoptotic functions [18].

It is also important to note that most of the p53 mutations involved in various cancers are in the DBD, as 50% of human cancers have inactivated p53 by mutation [19]. The oligomerization domain of p53, which is a function of its C-terminal domain, helps to regulate the cell cycle by MDM2, which efficiently binds and ubiquitinates p53, and induces proteasomal degradation. The mutations that delete or disrupt the function of the oligomerization domain of p53 have a low-binding affinity to MDM2, and therefore, affect the process of proteasomal degradation [20]. In addition, the C-terminal domain of p53 contains a nuclear export signal (NES) in which lysine residues are involved in the ubiquitination by MDM2. This helps the p53-MDM2 interaction at the N-terminal activation domain [21].

**Figure 2:**
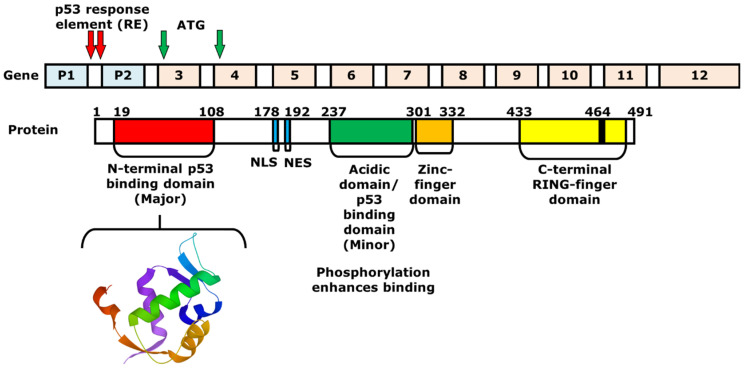
Structures of the *MDM2* gene and protein. The 3D structure of a portion of MDM2 protein (17–111) is shown below, which consists of the N-terminal p53-binding domain (19–108). The *MDM2* gene consists of two promoters and 10 additional exons. Between exons 1 and 2, or the first intron, there are two p53 response elements (REs), which are the two p53-binding sites. The REs are shown with red arrows. The full-length MDM2 protein p90 is produced by starting from ATG of exon 3, while the shorter protein, or p76, is synthesized with the start codon on exon 4.

MDM2 is a 491 amino acid regulator protein that consists of an N-terminal p53 binding domain, a nuclear localization signal (NLS), a NES, an acidic domain, a zinc-finger domain, and a C-terminal RING-finger domain. Similar to the binding of MDM2 at N-terminal activation domain of p53, the N-terminal p53-binding domain of MDM2 is an area that interacts with p53 (Figure 2). The NLS and the NES are essential for transporting MDM2 from the nucleus to the cytoplasm and vice versa. Within the acidic domain, residues are phosphorylated to induce the degradation of p53. Moreover, the C-terminal RING-finger domain functions as an E3 ubiquitin ligase that can induce the ubiquitination of p53 after p53-MDM2 interaction [22]. On the other hand, the zinc-finger domain, which is located next to the acidic domain, regulates the level of p53, as it interacts with ribosomal proteins that bind to the acidic domain and inhibits the degradation of p53 [23]. The *MDM2* gene that expresses the protein includes 12 exons. The two promoters P1 and P2 containing p53 response elements (RE) are shown with red arrows. The P1 and P2 promoters regulate the expression of two MDM2 proteins, p90 and p76, respectively. P90 is a full-length and fully functional protein that can bind to p53 at the p53-binding domain. In contrast, P76, which is shorter than p90, acts as a negative inhibitor of p90 and activates p53 because it lacks the p53-binding domain [12,24]. Next to the two promoters, there are 10 additional exons, in which the first two are involved in the translation of p90 and p76. Using ATG as the first start codon, p90 and p76 are translated into exon 3 and 4, respectively. The p76-Mdm2 inhibits the ability of p90-Mdm2 to destabilize p53 [25].

Many small molecules induce posttranslational modification of p53, including phosphorylation, acetylation, ubiquitination, sumoylation, neddylation and methylation, which prevent its degradation, and increase stabilization and transcriptional activity [26,27,28,29,30,31]. DNA-damaging agents, such as ultraviolet light (UV) radiation [32,33] and cisplatin compounds induce phosphorylation-dependent stabilization of p53 [34]. Among several phospho-acceptor sites on p53, the Ser15, Thr18, Ser20 are important for interaction with MDM2 [35,36]. A DNA-dependent acetylation of p53 modification facilitates chromatin remodeling and activates p53 target gene expression [37,38]. The acetylating residues of p53 are mainly located at its C-terminus. The most important of them are K370, K372, K373, K381, K382 and K386, which are acetylated by the highly related histone acetyltransferases p300 and cAMP response element binding (CREB)-binding protein (CBP) [39]. These same lysine residues are targeted by MDM2 for ubiquitylation [40,41]. As is the case for ubiquitination, there are two other ubiquitin-like proteins, small ubiquitin-like modifier (SUMO) and neural precursor cell expressed developmentally downregulated protein 8 (Nedd8), that can be conjugated to the lysine residues of p53 via the process of sumoylation and neddylation, respectively [42,43,44]. Mdm2, in addition to ubiquitination activity, can also promote the conjugation of Nedd8 to p53 as a part of its inhibitory function [30]. Nedd8 is covalently linked through its C-terminal carboxyl group to the *ε*-amino group of the lysine residues on target proteins in a ubiquitin-like manner [45]. The F-box domain protein (FBXO11) has been identified as an Nedd8 ligase for p53 and inhibits its transcriptional activity [46]. Methylation of p53 at lysine residues, like acetylation, stabilizes this protein, restricts it in the nucleus and enhances expression of *p21* gene expression [28]. On the other hand, the mono-methylation of p53 by Su(var), Enhancer of zeste, Trithorax 8 (SET8) and the SET domain-dependent methyltransferase subfamily (SMYD2) at K382 and K370, respectively, attenuate the transcriptional regulation activity of p53 [47,48]. In response to DNA damage, p53 can also be methylated at arginine residues and can affect p53 target gene expression [49].

## 2. Mechanism of p53 and MDM2-Binding

**Figure 3:**
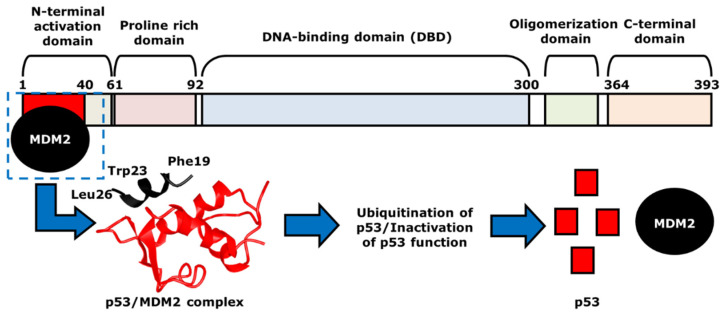
The mechanism of p53- and MDM2-binding. MDM2, indicated as black, binds to p53 at the N-terminal activation domain of p53, or the red area, to form p53/MDM2 complex. The two proteins are in direct contact via hydrogen bonding, with hydrophobic p53 residues Phe19, Trp23 and Leu26 that help such interaction. The structure of the p53/MDM2 complex, which is shown with the corresponding colors, black and red, leads to the ubiquitination and the proteasomal degradation of p53. This results in the inhibition of the transcriptional activity of p53.

In normal cells or cells that are repaired, the concentration of p53 is low because of a regulator protein called the murine double minute 2 (MDM2). As mentioned above, the *MDM2* gene is a negative regulator of p53, as it acts as a ubiquitin ligase [50,51]. It first physically interacts with p53 at TAD, which is amphipathic in nature. The binding pocket within the N-terminal p53-binding domain of MDM2 is hydrophobic in nature [52]. The p53 amino acid residues, including Phe19, Trp23, and Leu26, form the hydrophobic side of the amphipathic α-helix of the TAD. Although Phe19, Trp23, and Leu26 are not the only p53 residues that create the α-helix, these three amino acids play a key role in p53/MDM2 interactions [52]. Once the hydrophobic α-helix is formed, it contacts the hydrophobic binding pocket of MDM2, and thus, forms the p53/MDM2 complex via hydrogen bonding (Figure 3). During this interaction, Mdm2 induces the ubiquitination of p53 at the N-terminal activation domain, resulting in proteasomal degradation and inhibition of its transcriptional activity. One of p53 target genes is the cyclin-dependent kinase (CDK) inhibitor *p21*, which is a negative regulator of the cell cycle [53]. If the p53 is mutated, there will be no p21 activation, resulting the progression of the cell cycle even with damaged DNA [54]. As mentioned earlier, many of the human cancers inactivate p53 to allow for the continuation of cell cycle progression and cell survival. Thus, the p53-MDM2 interaction serves as an important focus area within cancer therapeutic studies. Current cancer therapeutics strive to increase levels of p53, by restoring the p53 function with the inhibition of its interaction with MDM2 or the degradation by MDM2. This strategy will prevent the survival and proliferation of tumor cells.

## 3. The Role of p53 in Cancer Chemotherapy

P53 is activated when DNA damage is sensed by the protein kinases ataxia telangiectasia mutated (ATM) and ataxia telangiectasia and Rad3-related (ATR), which activate the checkpoint kinases Chk2 and Chk1, respectively [55,56]. The ATM-Chk2 pathway is activated by the double-strand DNA breaks (DSB), while the ATR-Chk1 pathway is triggered by single-strand DNA breaks [57]. Both Chk1 and Chk2 induce the phosphorylation of p53, specifically, at serine (Ser)-15 and Ser-20 of the N-terminal activation domain, and thus, stabilize and activate p53 function [58,59]. As the levels of p53 increase, the activation of p53-dependent transcription of certain genes occurs, including *p21* and *MDM2* [60,61]. Similar to p53, p21 also acts as a tumor suppressor by regulating cell cycle and triggering apoptosis [62].

Under normal conditions, the complexes of CDK4/Cyclin D and CDK6/Cyclin D phosphorylate retinoblastoma tumor suppressor protein (Rb) and release the E2F transcription factor, which promotes the cell cycle to continue through the G_1_ phase into the S phase [63,64]. However, cells that express DNA damage increase p53-depnent expression of p21 causing cell cycle arrest at G_1_ by binding and inactivating CDK4-Cyclin D and CDK6/Cyclin D complexes. After the cell cycle arrest at G_1_ phase, the DNA damage can be repaired, and the cell cycle can progress through S phase. However, if the DNA damage cannot be fixed by the DNA repair machinery, there will be accumulation of DNA damage that can induce programmed cell death [65,66].

## 4. Strategies of Blocking the p53/MDM2 Interaction

The blocking of the p53/MDM2 interaction can be achieved by the following two mechanisms: first, by inhibiting the p53 and MDM2-binding, and second, by increasing the phosphorylation of p53. In the MDM2-binding mechanism, several small molecules have been discovered that bind to MDM2 by mimicking the p53-binding pocket residues Phe19, Trp23, and Leu26 [67,68]. Prohibiting the binding of MDM2 to p53 allows for the restoration of p53 tumor suppressor function. On the other hand, instead of directing binding to MDM2, compounds can also prevent the interaction by inducing the phosphorylation of p53. Several studies showed that DNA double-strand breaks induce the phosphorylation of p53 at Ser15 by ATM or DNA-protein kinase (DNA-PK) and at Ser20 by Chk2 [69,70,71,72]. Both of these are part of MDM2 binding [73]. The phosphorylation of p53 at these residues abrogates binding with Mdm2, and thus it spares p53 from ubiquitination and proteasomal degradation [70]. Besides these two ways, there are several other mechanisms that can inhibit the interaction, such as the degradation of MDM2. However, this paper will focus on compounds that have MDM2 binding abilities or lead to the phosphorylation of p53 as mechanisms of action. The MDM2 residues that are critical for its interaction with p53 protein are G58, D68, V75, and C77 [74]. Some of the many existing compounds that target p53-MDM2 interaction and that may be used in future cancer treatments are summarized in Table 1 [75].

### 4.1. AMG 232

#### 4.1.1. MDM2-Binding 

AMG 232 (C_28_H_35_Cl_2_NO_5_S, 568.6 g/mol, 2-[(3*R*,5*R*,6*S*)-5-(3-chlorophenyl)-6-(4-chlorophenyl)-3-methyl-1-[(2*S*)-3-methyl-1-propan-2-ylsulfonylbutan-2-yl]-2-oxopiperidin-3-yl]acetic acid) is an orally active MDM2 inhibitor [76,77]. By inhibiting MDM2 interaction, the degradation is inhibited, and its stability and transcriptional activity is increased. Similar to other MDM2 drugs, AMG 232 binds to the hydrophobic binding pocket of MDM2, at residues Phe19, Trp23, and Leu26. Specifically, the *m*-chlorophenyl, *p*-chlorophenyl, and C-linked isopropyl of AMG 232 interact with MDM2 [78].

#### 4.1.2. Treatments with AMG 232 

Ovarian Cancer: According to Sahin et al., AMG 232 induces p53 activation in ovarian cancer cells. For example, in their study, OVTOKO, OVMANA, and TOV-21G cells were treated with AMG 232. The results demonstrated that the p53 and its target gene *p21* was activated in all the tested cell lines. However, in OVTOKO and OVMANA, the cell lines with high MDM2 expression showed some resistance to the treatment [76]. Thus, although AMG 232 can be a drug for targeting the p53-MDM2 interaction and reactivation of p53, it may not be the best one due to the drug resistance in some cancer cell lines.

### 4.2. Chlorofusin

#### 4.2.1. MDM2-Binding

Chlorofusin (C63H99ClN12O19, 1364 g/mol, [(3*S*,4*S*,7*S*)-2-[3-[(2*S*,5*S*,8*S*,11*S*,14*R*,17*R*,20*S*,23*R*,26*R*)-11,14-bis(2-amino-2-oxoethyl)-5,20-bis[(1*R*)-1-hydroxyethyl]-8-methyl-17,23-bis(2-methylpropyl)-26-octyl-3,6,9,12,15,18,21,24,27-nonaoxo-1,4,7,10,13,16,19,22,25-nonazacycloheptacos-2-yl]propyl]-5-chloro-4-hydroxy-7-methyl-6,8-dioxospiro [4*H*-isoquinoline-3,2′-oxolane]-7-yl] butanoate) is a novel drug obtained from the fermentation of a microfungus belonging to genus Fusarium [79]. Discovered in 2001, it is a fungal metabolite and is found to inhibit the p53-MDM2 interaction via MDM2-binding. According to Clark and co-authors, Chlorofusin binds to the hydrophobic pocket within the N-terminal of MDM2 and disrupts the interaction with p53 at about 4.7 µM [80].

#### 4.2.2. Treatments with Chlorofusin

Liver Cancer: Although this compound can strongly bind to MDM2, and therefore, stabilize p53 activity, Chlorofusin does not affect HepG2, or the targeted liver cancer cell line. There is no cytotoxicity against these cells at 4 µM because the compound is inactive against Tumor Necrosis Factor (TNF) [80]. TNF, also known as TNFα, is a cytokine that has the ability to both induce and inhibit cancer cell growth. Many studies have focused on this cytokine as a way to promote cell death, including the ones involving Chlorofusin. Despite the fact that TNF-induced apoptosis is not being shown in HepG2 cells, Chlorofusin may still be a great candidate for improving cancer therapy, with its binding to MDM2 and targeting the p53-MDM2 interaction.

### 4.3. Flavonoids

Flavonoids are a group of natural products found in many fruits and vegetables [81]. They are often used as antioxidant, anti-inflammatory, anti-mutagenic, and anti-carcinogenic agents [81]. All flavonoids are compounds of a fifteen-carbon skeleton, with a heterocyclic ring in between two phenyl rings [82]. Based upon the type of functional groups attached to each ring and the degree of saturation, the flavonoids can be divided into several groups, such as flavones, flavonols, flavanones, flavanonols, flavanols, anthocyanins and chalcones [81].

#### 4.3.1. Chalcones

##### MDM2-Binding and Denaturation

Chalcones (C_15_H_12_O, 208.25 g/mol, (*E*)-1,3-diphenylprop-2-en-1-one), similarly to other flavonoids, are natural products found in many fruits and vegetables. They also have fifteen carbons in their chemical structure and provide anti-bacterial, anti-fungal and anti-inflammatory effects. All chalcones have an unsaturated carbonyl in between two phenyl rings and are known to prevent the interaction between MDM2 and p53 [83,84].

Derivatives of chalcones include A, B and C (Table 1), which can inhibit p53-MDM2 interactions either by directly binding to MDM2 or denaturing MDM2. There is also Chalcone D, but it does not affect the p53-MDM2 interaction. Because of this, chalcone D is used as a negative control in studies involving other chalcones [85]. Unlike D, derivatives A, B, and C have similar chemical structures, along with their ability to target MDM2. Compound A has a chlorine atom attached para to the carbonyl group on one phenyl ring and an acid group at the end of the other ring. Chalcone B, however, has an additional chlorine group on the same ring, while C has two methyl groups attached to the carbon atom α to the carboxylic acid functionality. Compounds A and C, which have only one chlorine group attached to the ring, interact with MDM2 by having the monosubstituted phenyl group and act like the tryptophane 23 (Trp23) residue. On the other hand, instead of directly binding to MDM2 like A and C, compound B denatures MDM2 [85]. This reveals the importance of functional groups, due to further chlorination at meta-position in addition to the p-chloro substitution, as in compound A. This possibly creates a steric hinderance, thus blocking the direct binding of compound B to MDM2 and forces it to inhibit the interaction using a different mechanism.

##### Treatments with Chalcones

Breast Cancer: Similar to chalcone derivatives A, B, and C, boronic chalcones can be used to inhibit cancer cells, specifically the breast cancer cell line MD Anderson Cancer Center Metastatic Breast cancer cell line 231 (MDA-MB-231). According to Kumar and colleagues, after treatment with these compounds, the IC_50_ values for growth inhibition in MDA-MB-231 cells were much lower than in normal breast cell lines Michigan Cancer Foundation 10A and 12A (MCF-10A and MCF-12A) [86]. This reveals that boronic chalcones are p53-dependent. Since in normal breast epithelial cells the level of p53 is very low, the effect of boronic chalcones is not seen. Even though boronic chalcones can successfully bind to MDM2 and prevent the degradation of p53 caused by MDM2, the compounds are less effective in normal cells, compared to cancer cells.

Skin Cancer (Melanoma): According to Kumar et al., the boronic chalcone derivatives can also be used to treat cancer cell line MDA-MB-435. When they published the article in the year 2003, the MDA-MB-435 cells were commonly used as breast cancer cells [86]. Therefore, the authors described that the chalcones were able to inhibit the growth of breast cancer cell lines, including MDA-MB-435. However, recent studies revealed that MDA-MB-435 was a melanoma cell line, instead of a breast cancer cell line [87]. Thus, in addition to breast cancer, these compounds are able to inhibit the growth of skin cancer as well.

#### 4.3.2. Tricetin (Flavones)

##### Phosphorylation of p53 

Tricetin (C_15_H_10_O_7_, 302.23 g/mol, 5,7-dihydroxy-2-(3,4,5-trihydroxyphenyl)chromen-4-one) is a flavonoid that reduces the likelihood of p53-MDM2 binding via the phosphorylation and stabilization of p53 [88]. This subgroup of flavones, in addition to the heterocyclic ring and two phenyl rings, has a double bond and a ketone group on the heterocyclic ring. The compound also consists of five hydroxyl groups, with two on one phenyl ring and three on the second phenyl ring. With this chemical structure, Tricetin is known to have a biological role as a metabolite in plants and is used as an antineoplastic agent to treat cancer, including breast cancer. This natural product phosphorylates p53 at serine 392 (Ser392 and Ser15, which increase the production and the activity of p53 as a tumor suppressor protein [89]. It can also induce cell cycle arrest at the DNA damage checkpoint between G_2_ and M phase, and apoptosis if DNA repair is compromised [88].

##### Treatments with Tricetin

Breast Cancer: It is important that Tricetin induces phosphorylation of p53 at Ser15 because it links p53 to an increased activation of ataxia telangiectasia-mutated (ATM). The *ATM* gene is a tumor suppressor within the phosphatidylinositol-3 kinase-like protein kinase (PIKK) family that plays an important role in breast cancer, similar to p53. When *ATM* is mutated, it may increase the risk for developing breast cancer [90]. However, in normal conditions, ATM is known to be involved in the repair DNA damages [91]. Thus, the increased activity from the phosphorylation by Tricetin induces p53 to become further stabilized. P53 stabilization then helps to decrease the interaction between p53 and MDM2, and thus, reduces the occurrence of p53 degradation by MDM2 [89].

### 4.4. Fluspirilene

#### 4.4.1. MDM2-Binding

Fluspirilene (C_29_H_31_F_2_N_3_O, 475.6 g/mol, 8-[4,4-bis(4-fluorophenyl)butyl]-1-phenyl-1,3,8-triazaspiro [4.5]decan-4-one) is an injectable antipsychotic drug used to treat chronic schizophrenic patients [92]. Even though the compound has been used in treating schizophrenia, recent studies show that Fluspirilene can also play a role in inhibiting the p53-MDM2 interaction.

#### 4.4.2. Treatments with Fluspirlene

Colon Cancer: Fluspirilene can disrupt the interaction by filling in the hydrophobic pocket and binding the site of MDM2. The compound can bind to MDM2 in two ways, in which the two fluorophenyl groups and the phenyl group can occupy either Leu26 or Phe19. For instance, the flurophenyl and phenyl groups can fit into the Leu26 and Phe19 pockets, respectively [93]. On the other hand, the orientation of the molecule can be changed with respect to the groups in the Phe19 and Leu26 pockets, respectively [93]. MDM2 is therefore prevented from binding to p53, as Fluspirilene binds to MDM2 instead of p53. Apart from this, Fluspirilene can also prevent cell growth in HCT116 cells, or the other cell lines associated with colon cancer, at the concentration of 10 µM. However, Fluspirilene performs its role as an inhibitor only if there is functional p53. This is demonstrated by Patil and co-authors, as there are significantly higher percentages of cell growth in HCT116-p53 knockout (−/−) cells than HCT116-p53 wild-type (+/+) cells [93]. Thus, Fluspirilene can be another great candidate for improving cancer therapy, as it is a p53-dependent inhibitor that targets the p53-MDM2 interaction and reduces cancer cell growth.

### 4.5. Hexylitaconic Acid

#### MDM2-Binding

Hexylitaconic acid (C_11_H_18_O_4_, 214.26 g/mol, 2-hexyl-3-methylidenebutanedioic acid) is a compound found in *Arthrinium* sp., a marine-derived fungus [94] prepared by racemic synthesis [95]. This compound can inhibit the p53-MDM2 interaction by competing against p53 and filling in the hydrophobic pocket of MDM2. Its chemical structure consists of two terminal carboxylic acid groups connected through two ethyl carbons with hexyl and a diene substitution, respectively. To understand the role of each functional group in relation to MDM2-binding, Tsukamoto and colleagues performed a study involving a comparison between the derivatives of hexylitaconic acid [94]. The following three derivatives were mentioned: one with the replacement of the acid with a methyl ester, second with reduction of the alkene group to an alkane, and third with both the replacement and reduction. Two additional compounds, itaconic acid and succinic acid, were also examined due to the similarity of their chemical structures, in that both have a functional dicarboxlic acid group. Compared to hexylitaconic acid, itaconic acid does not have the six-carbon hexyl chain substitution, while succinic acid lacked both the hexyl and the alkene substitutions. Using ELISA, they tested and discovered that these compounds did not bind to MDM2 as strongly and effectively as hexylitaconic acid. For example, the IC_50_ value for hexylitaconic acid is determined to be 50 µg/mL. When Tsukamoto and the others performed the test at that concentration for the three derivatives, the results showed that these molecules did not successfully block the p53-MDM2 interaction [94]. Thus, the functional groups of the hexylitaconic acid and its overall chemical structure play a crucial role in MDM2 binding and the inhibition of the interaction with p53.

### 4.6. Hoiamide D

#### 4.6.1. MDM2-Binding

Hoiamide D (C_35_H_58_N_4_O_7_S_3_, 743.1 g/mol, (2*R*,3*S*,4*S*,5*S*)-4-[[(4*S*)-2-[(4*R*)-2-[2-[(2*S*,3*R*,4*R*,5*S*,6*S*,7*R*)-4,6-dihydroxy-2-methoxy-3,5,7-trimethyldecyl]-1,3-thiazol-4-yl]-4-methyl-5*H*-1,3-thiazol-4-yl]-4-methyl-5*H*-1,3-thiazole-4-carbonyl]amino]-3-hydroxy-2,5-dimethylheptanoic acid) is an inhibitor of the p53-MDM2 interaction obtained from *Symploca* sp., a marine cyanobacterium. It is related to Hoiamides A through C because of its triheterocyclic structure and its ability to activate sodium channels [96]. Furthermore, Hoiamide C is closely related to Hoiamide D because they are both linear, while Hoiamide A and B have a macrocyclic ring structure [97]. Being linear, Hoiamide C has the same chemical structure as D, except that it has a terminal ester instead of a carboxylic acid group. However, despite of this, Hoiamide C does not affect the interaction as much as Hoiamide D [97], indicating the importance of the free carboxylic acid group. The other compounds, Hoiamide A and B, also do not show any significant effect [97], which could be attributed to their macrocycic structure. Therefore, compared to Hoiamides A through C, Hoiamide D can successfully prevent the interaction between p53 and MDM2, and can be used to target and promote apoptosis in cancer cells.

#### 4.6.2. Treatments with Hoiamide D

Non-Small Cell Lung Cancer: Hoiamide D, like other cyanobacterial-derived compounds, contains leucine and phenylalanine [98]. This can be an important consideration of the Hoiamide D structure because p53 also has similar amino acids, such as Leu26 and Phe19. Acting similarly to p53, Hoiamide D can fit into the hydrophobic binding pocket of MDM2 and block the p53-MDM2 interaction. This inhibition occurs at 4.5 µM, according to Malloy and colleagues [98]. In addition, Hoiamide D can halt cell growth, which has been tested with H460 or NCI-H460 cell lines. H460 is a non-small cell lung cancer cell line used in many studies involving human lung cancer. Cytotoxicity against this cell line is presented with Hoiamide D, after using the concentration of 40 µM [98]. Because the compound inhibits H460 cell growth and prevents MDM2 from binding to p53, Hoiamide D is an important natural product that can be used in further studies to improve lung cancer treatment.

### 4.7. Indole-3-Carbinol (I3C)

#### 4.7.1. Phosphorylation of p53

Indole-3-carbinol (C_9_H_9_NO, 147.17 g/mol, 1*H*-indol-3-ylmethanol), or I3C, is a compound obtained from vegetables within the genus *Brassica*, such as cauliflower, broccoli, and cabbage. It is a phytochemical that is produced from the breakdown of indole-3-ylmethylglucosinolate, more commonly known as Glucobrassicin, which are present in many cruciferous vegetables [99]. The product also reacts with itself and other metabolites to form conjugates, including indole-3-tryptophan and indole-3-carboxaldehyde [99]. I3C is known to prevent the p53-MDM2 interaction by p53 phosphorylation. This occurs at the N-terminal region, specifically Ser15 [100]. Such phosphorylation can block MDM2 from binding to p53, as all three of the hydrophobic binding pockets on MDM2, which are normally occupied by the residues Phe19, Trp23, and Leu26, need to be filled to successfully interact with p53.

#### 4.7.2. Treatments with Indole-3-Carbinol

Breast Cancer: Not only can it inhibit the p53-MDM2 interaction by the phosphorylation at Ser15, I3C also induces cell cycle arrest in breast cancer cells. For example, the MCF10A cell line, often considered as a non-cancerous human breast epithelial cell line [101], when treated with I3C experiences cell cycle arrest at the G_1_ phase [100]. The effect of I3C on cell cycle arrest has been shown through p53 because no cell cycle arrest occurred in the MCF10A cells that were stably expressed with dominant-negative p53 [100]. Thus, if p53 is present, I3C can induce phosphorylation of p53, which can then activate ataxia telangiectasia-mutated (ATM). Such activation of ATM, or a tumor suppressor within the phosphatidylinositol-3 kinase-like protein kinase (PIKK) family that repair damaged DNA, can lead to the inhibition of the p53-MDM2 interaction and the initiation of the G_1_ cell cycle [100]. A clinical trial on I3C has been completed in 2016. The report indicates that the trial did not complete. It stopped early and will not start again (ClinicalTrials.gov Identifier: NCT00033345).

### 4.8. Isokotomolide A (IKA)

#### 4.8.1. Phosphorylation of p53

Isokotomolide A (C_13_H_20_O_3_, 224.3 g/mol, (3*E*,4*S*)-4-hydroxy-5-methylidene-3-octylideneoxolan-2-one), also known as IKA, is a compound obtained from the plant *Cinnamomum* kotoense. Found in the leaves of the species, IKA is discovered to have the ability to inhibit the p53-MDM2 interaction by p53 phosphorylation [102]. It can phosphorylate p53 at the residue Ser15, which is one of the amino acids on p53 that directly interacts with MDM2 for binding. Because Ser15 is not available after introducing the compound, MDM2, with its one hydrophobic pocket not being filled, is not able to bind to p53. This prevents MDM2-mediated degradation of p53, which leads to an increased stability and activity of p53.

#### 4.8.2. Treatments with Isokotomolide A (IKA)

Lung Cancer: Not only can it prevent MDM2 from binding to p53, IKA can also inhibit lung cancer cell growth. For instance, after treatment of pulmonary epithelial cell line A549 with IKA, the cell proliferation of these cells is inhibited [103]. Chen and coauthors have discovered the IC_50_ for IKA being 4.4 µM, and that 10 µM is needed for complete cell growth inhibition [102]. In addition, similar to other small molecules that target the p53-MDM2 interaction, IKA is able to promote cell cycle arrest and apoptosis. The cell cycle arrest occurs between phases G_0_ and G_1_ and is shown to have a significant effect at 6 h [102]. Therefore, IKA is another natural product that can both disrupt the p53-MDM2-binding by p53 phosphorylation and induce cell cycle arrest and apoptosis.

### 4.9. Lithocholic Acid (LCA)

#### 4.9.1. MDM2-Binding

Lithocholic Acid (C_24_H_40_O_3_, 376.6 g/mol, (4*R*)-4-[(3*R*,5*R*,8*R*,9*S*,10*S*,13*R*,14*S*,17*R*)-3-hydroxy-10,13-dimethyl-2,3,4,5,6,7,8,9,11,12,14,15,16,17-tetradecahydro-1*H*-cyclopenta[a]phenanthren-17-yl]pentanoic acid), also known as LCA, is an endogenous steroidal bile acid that binds to MDM2 and blocks the p53-MDM2 interaction [104]. As a secondary bile acid, LCA is commonly found in the bile and is used to solubilize fats for absorption. This compound consists of three cyclohexanes and one cyclopentane, which is attached to a carboxylic acid. It is important to note that high levels of LCA can increase the risk of developing cancer, including colon cancer.

#### 4.9.2. Treatments with Lithocholic Acid

Colon Cancer: Although at a high concentration LCA is toxic and carcinogenic, studies have shown that it also can reduce the risk of cancer development by blocking the p53-MDM2 interaction and inducing apoptosis. For example, as a dual inhibitor, LCA can bind to both MDM2 and MDM4. MDM4, also known as MDMX, is similar to MDM2 with its role in the regulation of p53 levels [104]. MDM4 binds to the compound slightly stronger than MDM2 since the dissociation constants of LCA for MDM4 and MDM2 are 15.4 µM and 66.0 µM, respectively [104]. Despite this difference, LCA can successfully bind to MDM2, and therefore, increase the stability of p53. However, the strength of the binding is weakened after changing and modifying the chemical structure of LCA. In addition to binding, LCA can induce apoptosis in colon cancer cells, such as in HCT116, the human colorectal carcinoma cell line. Treatment of HCT116 cells with LCA at 300 µM concentration increases caspase 3 and 7 activation, which are the apoptosis markers [104]. Such an apoptotic effect in HCT116 can be explained by the binding of LCA to MDM2, as well as to MDM4, resulting in increased levels of p53 [104].

### 4.10. Nutlins

#### 4.10.1. MDM2-Binding

The Nutlins are a family of MDM2 inhibitors discovered by Vassilev and others, and consist of Nutlin-1, Nutlin-2, and Nutlin-3. They are small molecules that inhibit the formation of the p53/MDM2 complex via MDM2-binding, and thus increase p53 protein stability through post-translational modification [105,106]. The core structure of the Nutlins is *cis*-imidazoline. This structure was discovered by in vivo experimental screening, in which the purpose was to find small molecule inhibitors of MDM2 through multiple chemical modifications [67]. With the structure of *cis*-imidazoline, the Nutlins are shown to be the first small molecules able to achieve inhibition by specific binding interactions between p53 and MDM2 [67]. This is accomplished by adding functional groups, such as halogenated phenyl rings that mimic the hydrophobic p53 amino acid residues Phe19, Trp23, and Leu26. Out of the Nulin family, Nutlin-3 has been shown to inhibit the interaction at a greater level of specificity than the others, as it induces the stabilization and activation of p53 [107]. Because of this, Nutlin-3 is the one that is most used and evaluated in cancer therapeutic studies.

#### 4.10.2. A. Nutlin-1 and Nutlin-2

Nutlin-1 (C_32_H_34_Cl_2_N_4_O_4_, 609.548 g/mol, 1-[4-[4,5-bis(4-chlorophenyl)-2-(4-methoxy-2-propan-2-yloxyphenyl)-4,5-dihydroimidazole-1-carbonyl]piperazin-1-yl]ethanone) contains three functional groups that mimic certain residues of p53 when binding to MDM2 [108]. These include two halogenated phenyl rings and an isopropoxy group on the methoxy-phenyl ring. The halogen on the two phenyl rings is chlorine, which helps rings to substitute the p53 residues Leu26 and Trp23. The last residue, Phe19, is replaced by the isopropoxy group [109]. On the other hand, although the two halogenated phenyl rings are maintained, the halogen used in Nutlin-2 (C_31_H_34_Br_2_N_4_O_4_, 686.4 g/mol, [(4*S*,5*R*)-4,5-bis(4-bromophenyl)-2-(2-ethoxy-4-methoxyphenyl)-4,5-dihydroimidazol-1-yl]-[4-(2-hydroxyethyl)piperazin-1-yl]methanone) is bromine instead of chlorine. In addition, the isopropoxy group that substitutes Phe19 is replaced with an ethyl ether group. Similar to Nutlin-1 and Nutlin-3, the bromo-substituted phenyl rings also fill the Leu26 and Trp23 binding pockets, while the ether substituent is in the Phe19 pocket [109].

#### 4.10.3. B. Nutlin-3

Nutlin-3a verses Nutlin-3b: Nutlin-3a and Nutlin-3b are the two enantiomers of Nutlin-3, Nutlin-3a is (−)-Nutlin-3 and Nutlin-3b is (+)-Nutlin-3. Nutlin-3a is 150-fold more potent as an inhibitor of p53-MDM2 interactions than Nutlin-3b, which does not effectively bind with MDM2 [105]. For example, Nutlin-3b binds to MDM2 with an approximately 200-fold lower affinity than Nutlin-3a, which indicates stereoselectivity [110]. It has been discovered that MDM2 favors Nutlin-3a and not Nutlin-3b because of the basins of attraction, which is the area of attraction around MDM2 [111]. Although Nutlin-3a and Nutlin-3b both have two basins of attraction around MDM2, including CTER on one side of the binding site and NTER on the other, the CTER on Nutlin-3a is closer to its binding site than Nutlin-3b [112]. With such a basin, Nutlin-3a can bring the MDM2 closer to its binding site and thus more effectively bind to MDM2 than its mirror image.

In addition to the location away from the binding site, the CTER basin of Nutlin-3b extends more towards the residue lysine 70 (K70) on MDM2. Further investigation revealed that K70 plays a role in determining the affinities of Nutlin-3a and Nutlin-3b. The mutation at K70 results in the disappearance of a basin in each isomer, such as the NTER basin in Nutlin-3b and the CTER basin in Nutlin-3a [112]. It is not only the NTER basin that is impacted, but the CTER of Nutlin-3b is also impacted, as it has a decreased the extension towards K70. On the other hand, even though CTER disappears after the mutation, the remaining basin, or the NTER basin, is not affected. Due to the differences in the effects on the basin in each isomer, with one decreasing its area of attraction and the other having no effect, Nutlin-3b has a lower binding affinity for MDM2. Having such low affinity, Nutlin-3b is the inactive enantiomer, which could be used in experiments as a control for examining off-target drug effects [113].

Nutlin-3a consists of the same functional groups as Nutlin-1, which includes two chlorophenyl rings and an isopropoxy group on a methoxy-phenyl ring. They are maintained because the rings fill the binding pockets of Leu26 and Trp23 completely, and the Phe19 pocket is better filled with Nutlin-1 than Nutlin-2 [114]. With the same binding sites, these molecules can bind to MDM2, instead of p53 interacting with MDM2. Moreover, with these functional groups, Nutlin-3a serves as the better molecule, out of Nutlin-1, Nutlin-2, and Nutlin-3b, for inhibiting the interaction between MDM2 and p53. This is due to the lower IC_50_ of 90 nM, while the IC_50_ of Nutlin-1 and Nutlin-2 are 260 nM and 140 nM, respectively [67]. By inhibiting this interaction, p53 does not go through degradation, and thus performs its function as an inducer of cell cycle arrest and apoptosis.

#### 4.10.4. Treatments with Nutlin-3a

Colon Cancer: Nutlin-3 can increase the levels of p53, which can then activate its function and slow the DNA repair process in colon cancer cells [115]. This induces the DNA damage response during the S phase of the cell cycle as γH2AX, a marker of DNA damage and repair that increases after treatment with Nutlin-3 [116]. However, Nutlin-3 cannot be used alone for treating colon cancer because the number of γH2AX decreases after using multiple doses. Instead, doxorubicin, or topoisomerase II-targeting drugs can be used in addition to Nutlin-3 to further increase the levels of γH2AX [117]. The combination effects include a high level of production of γH2AX and an irreversible growth inhibition of colon cancer cells [115].

Lung Cancer: Treating lung cancer involves radiotherapy, in which ionizing radiation (IR) suppresses the growth of the lung cancer cells via apoptosis [118]. Further investigations reveal that such inhibition is achieved by activating p53 [118]. To upregulate p53, in addition to radiation therapy, the treatments involve the use of Nutlin-3. The combined treatment is shown to enhance the cytotoxicity of ionizing radiation (IR), which can help sensitize the cells and induce cell death. Thus, Nutlin-3 in combination therapy sensitizes many cancer cell types, but shows less effect in those with poor *MDM2* expression [107,119].

Osteosarcoma (OS): Nutlin-3 stabilizes and activates p53, which induces G_1_ and G_2_ phase cell cycle arrest and apoptosis in osteosarcoma (OS) cells. However, such apoptosis and the inhibition of growth occurs in OS cells containing wild-type p53, for example, U-2 OS cells [120]. On the other hand, MG63 and SaOS2 cells with mutant and null p53, respectively, show no Nutlin-3-dependent growth inhibition [120]. This suggests that the use of Nutlin-3 is limited by the p53 status, in which Nutlin-3 is only effective for wild-type p53. Thus, the drug Oridonin can be used in OS cells with wild-type p53, in addition to Nutlin-3. Oridonin is a potent cytotoxic agent and is known to have no impact on the levels of p53 [120,121]. However, the combination of Oridonin and Nutlin-3 induces apoptosis [120]. Using both Oridonin and Nutlin-3 can, therefore, create a synergistic effect and further inhibit cell growth.

Nasopharyngeal carcinoma (NPC): p53 mutations are shown to be rare in nasopharyngeal carcinoma (NPC) [122]. This indicates that the p53-MDM2 interaction could be a therapeutic target for NPC cells [123]. After testing the effects of Nutlin-3 on NPC and nasopharyngeal epithelial (NPE) cell lines, it has been revealed that the drug inhibits the p53-MDM2 interaction more strongly in NPC cells (C666-1) than in NPE cells (NP69 and NP460) [124]. Moreover, current treatments for NPC involve chemotherapy, using cisplatin and 5-fluorouracil [125]. It has been shown that Nutlin-3 works synergistically with cisplatin. However, further studies show that there is a reduced sensitivity of C666-1 cells with long-term treatment with Nutlin-3 [124]. Although there are limitations, Nutlin-3 is an anticancer drug with great potential and may be used in the future to enhance the efficacy of chemotherapy for NPC.

### 4.11. Idasanutlin (RG7388)

#### 4.11.1. MDM2-Binding

Idasanutlin (C_31_H_29_Cl_2_F_2_N_3_O_4_, 616.5 g/mol, 4-[[(2*R*,3*S*,4*R*,5*S*)-3-(3-chloro-2-fluorophenyl)-4-(4-chloro-2-fluorophenyl)-4-cyano-5-(2,2-dimethylpropyl)pyrrolidine-2-carbonyl]amino]-3-methoxybenzoic acid), also known as RG7388, inhibits the p53-MDM2 interaction by binding to MDM2. As suggested by the name, Idasanutlin is a second generation nutlin that has been designed to increase the drug efficacy of the first generation nutlins [126,127].

#### 4.11.2. Treatments with Idasanutlin

Acute Myeloid Leukemia (AML): With an increased efficacy, Idasanutlin, as of 2018, is the only MDM2 antagonist that has entered phase III clinical trials [128]. Passing the phase II clinical trials, the drug shows more potent efficacy and fewer side effects compared to the first generation nutlins. However, the phase III clinical trial of idasanutlin was completed and a number of patients with dose-limiting toxicities were observed and there were a number of participants with adverse effects. A new clinical trial phase I/II begam in July 2019 and currently is underway (ClinicalTrials.gov Identifier: NCT04029688). At present, researchers are investigating the effects of Venetoclax (ABT-199), in addition to Idasanutlin on AML patients because Venetoclax is a BCL-2 inhibitor that has a synergistic effect with Idasanutlin [106].

Another approach to p53-mediated cell cycle arrest and apoptosis can be the reactivation of p53. For example, in diffuse intrinsic pontine gliomas (DOPGs) where p53 is dysregulated by *Protein Phosphatase, Mg^2+^/Mn^2+^ Dependent 1D* (*PPM1D*) gain-of-function mutations [129], the Idasanutlin has been shown to selectively inhibit the proliferation of the TP53 wild-type/PPM1D mutant DIPG cell lines in a dose- and time-dependent manner. It was also noted that the anti-proliferative effects of Idasanutlin were p53-dependent [130].

### 4.12. SAR405838 (MI-77301)

#### 4.12.1. MDM2-Binding

SAR405838 (C_29_H_34_Cl_2_FN_3_O_3_, 562.5 g/mol, (2′*R*,3*R*,3′*S*,5′*S*)-6-chloro-3′-(3-chloro-2-fluorophenyl)-5′-(2,2-dimethylpropyl)-*N*-(4-hydroxycyclohexyl)-2-oxospiro [1*H*-indole-3,4′-pyrrolidine]-2′-carboxamide), also known as MI-77301, is a novel drug that inhibits p53–MDM2 interaction by binding to MDM2. Similarly to other compounds that bind to MDM2, SAR405838 has three binding pockets similar to MDM2. Because of this, SAR405838 can compete against p53 and bind to MDM2. Not only by using the binding pockets, but SAR405838 can also interact with MDM2 using the Cl atoms. For example, SAR405838 consists of chlorine atoms in the oxindole groups [131]. SAR405838 additionally interacts with MDM2 via hydrophobic interactions between the Cl atoms of SAR405838 and MDM2 [131]. Moreover, further interaction occurs between the compound and the N-terminal region of MDM2, which does not occur in the p53-MDM2 interaction. Given the several contact areas between SAR405838 and MDM2, the SAR405838-MDM2 complex forms, which then allows p53 to perform its role as a tumor suppressor. However, it is important to note that there are some limitations to the drug treatment. For instance, even though SAR405838 can inhibit cancer cell growth by binding to MDM2 and increase p53 levels, the effectiveness of the drug is reduced when there is p53 mutation or deletion. This reveals that SAR405838 is a p53-dependent drug [131]. Despite some limitations, SAR405838 is a compound that may be useful for future cancer therapy with its effects of inhibiting cancer cell growth.

#### 4.12.2. Treatments with SAR405838

Colon Cancer: According to Wang et al., SAR405838 inhibits tumor growth in cancer cells [131]. In their experiment, the authors administered a 200 mg/kg of SAR405838 orally each day to mice bearing HCT-116 cells tumor. The treatment was performed for approximately 3 weeks. The results showed that SAR405838 was able to inhibit the growth of colon cancer xenografts in mice [131].

Prostate Cancer: SAR405838 is also able to inhibit the growth of prostate cancer cells, specifically, the LNCaP (Lymph Node Carcinoma of the Prostate) cells. According to Wang et al., 100 mg/kg of SAR405838 was administered orally to mice daily for 4 weeks [131]. The results revealed drug-induced growth inhibition in prostate cancer cells. In addition, the authors demonstrated that using 200 mg/kg would induce 80% regression [131]. Even though twice the amount of the drug was used to inhibit growth, the experiment showed no toxicity in mice.

**Table 1:** List of compounds that target p53-MDM2 interaction, either via MDM2-binding or phosphorylation of p53.

Compound.	Mechanism of Inhibition	Cancer Type	Structure	References
AMG 232	MDM2-binding	Ovarian Cancer	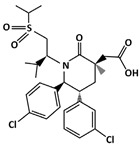	[77]
Chlorofusin	MDM2-binding	Liver Cancer	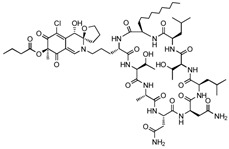	[79]
*Flavonoids*				
Chalcones: Chalcone A Chalcone B Chalcone C	MDM2-binding and denaturation	Breast Cancer Skin Cancer (Melanoma)	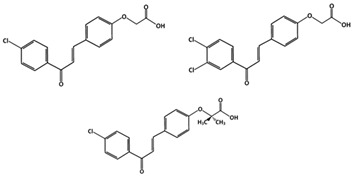	[83]
Tricetin (Flavones)	Phosphorylation of p53	Breast Cancer	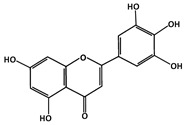	[84]
Fluspirilene	MDM2-binding	Colon Cancer	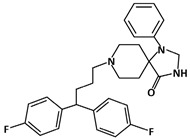	[91]
Hexylitaconic Acid	MDM2-binding	Not reported	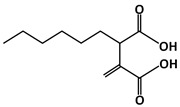	[95]
Hoiamide D	MDM2-binding	Non-Small Cell Lung Cancer	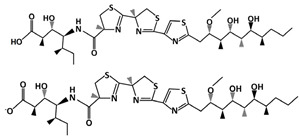	[96]
Indole-3-carbinol (I3C)	Phosphorylation of p53	Breast Cancer	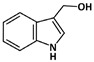	[99]
Isokotomolide A (IKA)	Phosphorylation of p53	Lung Cancer	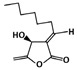	[100]
Lithocholic Acid (LCA)	MDM2-binding	Colon Cancer	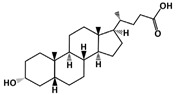	[104]
*Nutlins*				
Nutlin-3a	MDM2-binding	Colon Cancer Lung Cancer Osteosarcoma (OS)Nasopharyngeal carcinoma	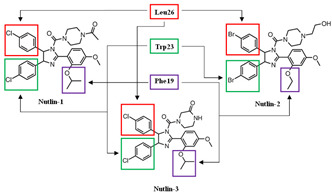	[105]
Idasanutlin (RG7388)	MDM2-binding	Acute Myeloid Leukemia (AML)	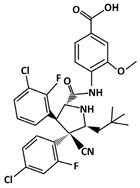	[127]
SAR405838 (MI-77301)	MDM2-binding	Colon Cancer Prostate Cancer	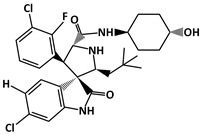	[131]

## 5. Conclusions

The structural features and roles of p53 and MDM2 reveal the importance of targeting the p53-MDM2 interaction. Disrupting this interaction can promote cell cycle arrest and apoptosis by allowing p53 to perform its role as a tumor suppressor. To increase the p53 levels, and thus induce cancer cell death, researchers have discovered compounds that prevent the direct contact of MDM2 with p53. Compounds, such as the Nutlins and the Chalcones, can either bind to MDM2 or enhance the phosphorylation of p53. The experimental results demonstrate that the drugs targeting MDM2 and p53 interaction are effective in p53 activation, as the growth of different cancer cells has been inhibited and apoptosis has been induced. Although much research has been produced in this area to develop inhibitors of p53-MDM2 interaction, only two compounds, Idasanutlin and I3C have progressed to clinical trials. While the success of these two compounds has still to be determined, there is a critical need to develop small molecules that may inhibit the interaction of p53-Mdm2 and can be developed as potent therapeutic agents with lower or nonclinical toxicity. Although most of the compounds could not make it to the clinical trials due to various reasons, many of them can serve as critical lead compounds for the development of more refined and efficacious inhibitors of p53-Mdm2.
